# Uncovering the invisible giant: Amyloid beta plaques and their proposed association with waste removal in Alzheimer-affected human hippocampus

**DOI:** 10.21203/rs.3.rs-9337412/v1

**Published:** 2026-04-30

**Authors:** Ruth Fabian-Fine, Abigail G. Roman, Melanie J. Winters, Kyleena J. Lathram, Carly H. Bennett, Luwago K. Kipingi, Sondre M. Brännare-Gran, Abigail E Whitley, Chloe M. Paul, Lydia M. Altman, Ian C. Carrillo, Finn M. Joyce, Lydia A. Kragh, Theodore J. McKnight, Calum J. Reding, Leaf J. D. Reiderer, Lesley J. Rivera, Hannah A. Steen, Adam L. Weaver

**Affiliations:** 1 Department of Biology, Saint Michael’s College, Colchester, VT 05439, USA; 2 Students participating in the 2025 Developmental Biology Course-Based Undergraduate Research Experience (CURE) course.

**Keywords:** Aquaporin 4, Myelin, Tanycyte, Convective flow, Neurodegeneration

## Abstract

According to the prevalent ‘Amyloid Hypothesis,’ the underlying cause for neurodegeneration in Alzheimer Disease (AD) is attributed to the accumulation of misfolded Amyloid ß and tau protein in the form of extracellular sticky plaques and neurofibrillary tangles, respectively. These protein accumulations are thought to be caused by impaired waste removal. In an alternative hypothesis, we have proposed the existence of an extensive glial canal system that is likely formed by myelinated aquaporin-4 (AQP4)-expressing tanycytes and removes cellular waste from the hippocampal formation. Here, we demonstrate that tanycyte-derived waste-internalizing receptacles are immunoreactive for Aß and emanate from specialized nucleus-like organelles in the following referred to as ‘tanysomes.’ Utilizing RNA-scope in situ hybridization, we demonstrate that these receptacle-forming tanysomes express RNA for AQP4 and the Aß-related genes, amyloid precursor protein, and presenilin-1. These findings suggest that Aß is likely synthesized where receptacle formation is observed and that Aß may play an important structural role in receptacle formation. In AD-affected hippocampus, excessive amounts of Aß-immunoreactive waste receptacles emerge from tanysomes and have the appearance of plaques in Aß-immunolabeled hippocampus. Moreover, we demonstrate that the same receptacle-forming organelles exhibit strong immunolabeling for hyperphosphorylated tau protein in AD-affected tissue. We postulate that both proteins may play important structural roles in waste uptake and that hypertrophic swelling of impaired tanycytes in AD-affected brain may be due to obstructions of this extensive interconnected glial canal system.

## Introduction

Over 50 million people worldwide are believed to be affected by progressive neurodegenerative diseases (Asher and Priefer, 2022, Breitner, et al., 2022, Carlsson, 2008, Cummings, et al., 2021, Gustavsson, et al., 2023, Huang, et al., 2019, Sathyanarayana Rao and Jagannatha Rao, 2009, Tatulian, 2022). One of the most prevalent forms of neurodegeneration is associated with Alzheimer disease (AD) that affects roughly 6.9 million USA citizens over the age of 65, thus ranking within the leading causes of death (2024). However, despite massive international research efforts, the underlying causes for AD remain elusive. Our lack of insight into the cellular processes that trigger neurodegeneration may help explain the repeated setbacks the pharmaceutical industry has encountered in clinical trials (Asher and Priefer, 2022, Breitner, Dodge, Khachaturian and Khachaturian, 2022, Carlsson, 2008, Cummings, Lee, Zhong, Fonseca and Taghva, 2021, Huang, Shen and Zhao, 2019, Sathyanarayana Rao and Jagannatha Rao, 2009, Tatulian, 2022).

Over the past several decades, the ‘amyloid hypothesis’ has been the prevalent proposition regarding the underlying causes of AD. One prevalent theory postulates that the presence of misfolded, ‘amyloid beta (Aβ) plaques’ in the brain of AD decedents may be the result of failed waste removal from the brain(Nedergaard, 2013, Pedersen, et al., 2023a, Pedersen, et al., 2023b)

Based on the amyloid hypothesis, these protein aggregates obstruct intra- and extracellular spaces thereby triggering neurodegeneration. This prevailing theory led to drugs designed to clear Aβ and hyperphosphorylated tau protein from the brain using monoclonal antibodies (2024, Asher and Priefer, 2022, Breitner, Dodge, Khachaturian and Khachaturian, 2022, Carlsson, 2008, Cummings, Lee, Zhong, Fonseca and Taghva, 2021, Gustavsson, Norton, Fast, Frolich, Georges, Holzapfel, Kirabali, Krolak-Salmon, Rossini, Ferretti, Lanman, Chadha and van der Flier, 2023, Huang, Shen and Zhao, 2019, Sathyanarayana Rao and Jagannatha Rao, 2009, Tatulian, 2022). However, these approaches have yielded limited cognitive benefits to patients with numerous trials failing in the clinical phase (Asher and Priefer, 2022). In addition to the suffering experienced by affected individuals and their families, the monetary implications of these failed drug trials are staggering. With an estimated annual cost of US$ 300 billion in healthcare-related expenses (2024), these numbers highlight the paramount importance to re-evaluate past and current research findings and therapeutic approaches.

Why have we been unable to illuminate the underlying causes for neurodegeneration despite (i) massive international research undertakings, (ii) advanced scientific knowledge, and (iii) increasingly sophisticated biomedical technologies?

With these progress limitations in mind, we argue that all assumptions regarding AD etiology be critically revisited, and basic characterization of healthy and AD-affected cellular changes be re-investigated. In this study, we have revisited cellular structure utilizing high-resolution cell imaging, RNA-scope in situ hybridization, immunohistochemistry, and correlative light- and electron microscopy to investigate where Aβ-related genes are expressed in relation to Aβ plaques. Utilizing this knowledge, we studied how plaque-formation starts and how these plaques grow in AD-affected human hippocampus.

Due to the prevalence of rodent-models in AD research, we have also investigated mouse and rat tissue. Based on the findings presented here, we propose that the answer to the above-stated question may be misidentification of cellular structure. Here, we provide a novel hypothesis that Aβ plaques in AD-affected hippocampus may not represent accumulations of misfolded Aβ protein deposits (Kepp, et al., 2023), but may show abnormal proliferation and swelling of proposed waste-internalizing receptacles that are formed by a recently-discovered glial canal system (Fabian-Fine, et al., 2024). We show that the Aβ-immunolabeled receptacles express the Aβ-related genes amyloid precursor protein (APP) and presenilin-1 (Pres1). Based on compelling structural evidence provided here, we postulate that the functional significance of Aβ along waste receptacles is likely their structural stabilization to prevent the collapse of these membranous receptacles during waste intake.

The preliminary findings presented here provide a new perspective into underlying causes for AD-related neurodegeneration. We encourage other researchers to exhaustively investigate and critique our hypotheses.

## Materials & Methods

### Brain tissue

The human brain tissue investigated here was obtained by Dr. John DeWitt (University of Vermont Medical Center) in the context of a previously published study (Fabian-Fine, Weaver, Roman, Winters and DeWitt, 2024). In brief, the tissue originated from autopsy examinations of four decedents at the University of Vermont Medical Center. The tissue was obtained with full written consent from the next of kin for biomedical research, diagnostic, and teaching purposes, and was fixed (see below) immediately after brain removal. All processes were in accordance with Vermont State law, the Health and Human services regulation 45 CFR 46.102(e), and the University of Vermont regulations regarding human subjects research. Tissue from two 86-year-old decedents (post-mortem intervals of 15 and 16 h) showed accumulations of amyloid β plaques and phosphorylated tau tangles, with ABC scores consistent with intermediate (A2B2C1) and high (A3B3C2) burdens of AD neuropathologic change. Tissue obtained from two 76- and 35-year-old decedents (45- and 14-hours post-mortem intervals) were negative for AD neuropathologic change. The mouse brain originated from three adult (15-month-old) female wildtype mice (Cdh5-GCaMP8 strain). For ethical reasons and to avoid unnecessary animal sacrifices, the brains used in this study were obtained from surplus tissue. To prevent animal suffering, the animals were euthanized by a fully trained and licensed technician using Euthasol (Virbac). The tissue was obtained freshly upon dissection of the animals and fixed in ice-cold 4% paraformaldehyde in phosphate buffered saline 0.1 M, pH 7.4. IACUC approval was granted under PROTO202200018 (University of Vermont) and IACUC-2024–001 Fabian-Fine (Saint Michael’s College). The rat brain was obtained from two healthy three-month-old male Sprague Dawley control rats and embedded into Araldite as described below.

### Immunohistochemistry

Brain tissue processed for immunolabeling was fixed in freshly prepared 4% paraformaldehyde (EMS 15710) overnight in the fridge. The tissue was embedded in 4% Agarose (Sigma # A 9539) and sectioned into 70 μm vibratome sections using a Leica S 1000 vibratome. The sections were washed 4×5 min in 0.1 M phosphate buffered saline pH 7.4 (PBS) prior to immersion in incubation and blocking medium (IBM; 0.25% Bovine Serum Albumin (Sigma A4503), 5% Normal goat serum (Sigma G9023) in 1% Triton-X/PBS) for 20 min. The sections were incubated with the primary antibodies overnight at 6 °C. The primary antibodies utilized were rabbit anti-AQP4 (BiCell #20104), Mouse anti-APP-C99; Sigma Aldrich MABN380, goat-anti mouse anti-Vimentin (AMF-17b-s DSHB, IOWA) and mouse anti-Myelin 6–4H2 DSHB, IOWA, mouse anti-APP-C99; Sigma Aldrich MABN380, and rabbit anti-Presenilin-1 ZRB1614 Sigma Aldrich at dilutions of 1:100 in 10% IBM and 90% PBS.

Subsequently the preparations were washed in PBS 5×10 min, incubated in IBM (20 min) and incubated in the secondary antibodies overnight 6 °C. The secondary fluorochrome-coupled antibodies were Cy3 goat anti-rabbit, Jackson ImmunoResearch Laboratories 111-165-003, and FITC goat anti-mouse Jackson ImmunoResearch Laboratories 115-096-072. Hoechst Blue nuclear stain was used to visualize nucleic acids at a dilution of 1:3000 in PBS for 20 min. After thorough washing in ice cold PBS for 90 min, the vibratome sections were mounted on glass slides and embedded in Mowiol (Sigma 81381). To avoid bleaching of the secondary antibodies all steps were performed in the absence of sunlight.

The sections were analyzed using a confocal Zeiss AxioImager MZ with Apotome.

### Fluorochrome uptake experiments in living mouse brain

To investigate whether waste swell-bodies and associated receptacles in living mouse brain internalize Cy3 fluorochromes, we have exposed freshly dissected mouse brain to these fluorochromes as described previously (Fabian-Fine, et al., 2025).

### Immunoperoxidase stain

Immunoperoxidase stain was carried out in the histology lab of the University of Vermont Medical Center. For Aβ and Tau protein using paraffin-embedded 5 μm-thick brain sections. The detailed procedure using the Leica Bond-3 auto staining system was described previously (Fabian-Fine, Weaver, Roman, Winters and DeWitt, 2024). The primary antibodies used were β Amyloid 1–42 (mOC 64, AbCam) and Tau AT8 directed against Phospho-Tau Serine 202 and Threonine 205 (AT8, Thermo Scientific # MN1020).

### Tissue preparation for electron microscopy

Brain tissue that was processed for electron microscopy was fixed in freshly prepared 4% paraformaldehyde containing 2.5% glutaraldehyde (EMS 16019) in PBS. The brains were embedded in 4% agarose and sectioned into 70–300 μm vibratome sections. After washing in PBS the sections were post-fixed for 30 min in 1.0% osmium tetroxide (Electron Microscopic Sciences 19150). After rinsing in PBS (2×5 min), the sections were dehydrated using a graded series of ethanol according to standard electron microscopy protocols. The preparations were transferred into propylene oxide (Electron Microscopic Sciences 20401), slowly infiltrated with Araldite (Electron Microscopic Sciences 13900), and polymerized overnight at 60 °C, according to manufacturer’s instructions.

### Ultrathin sectioning

The araldite embedded tissue was trimmed and sectioned with an 8-mm Diatome histo-knife using a Leica Ultracut E at a thickness of 65-nm. To prevent wrinkles, we utilized wooden toothpicks that were soaked with chloroform (Electron Microscopic Sciences, #12540) and held over the sections at approximately 2 mm distance to stretch them. It is important not to touch the sections with the toothpicks as it will damage them. The sections were collected on pioloform-coated single-slot copper or nickel grids (EMS# G2010CU). Contrasting was carried out using aqueous 1.5% uranyl acetate (6 min) and Reynold’s lead citrate (5 min) according to standard protocols. Electron microscopic examination was conducted using a JOEL 1400 electron microscope operated at 80 kV.

#### RNAscope in-situ hybridization

We utilized *in*-*situ* hybridization for the detection of AQP4, Amyloid Precursor Protein and Presenilin-1 gene expression as described earlier using formalin-fixed 5-μm paraffin embedded human hippocampal sections (Fabian-Fine, Weaver, Roman, Winters and DeWitt, 2024). The complementary probes were designed by Bio-Techne. The paraffin sections were obtained from the histology lab of the University of Vermont Medical Center. These experiments were carried out in the University of Vermont Center for Biomedical Shared Resources.

### Controls

Overall RNA integrity was assessed using *in situ* hybridization for the low level expressed ‘housekeeping’ gene peptidylpropyl isomerase B. For negative controls, we utilized the bacterial gene dapB. We routinely assessed RNA integrity (PPIB-positive and dapB-negative) for all samples.

### Luxol H&E staining of autopsy tissue

This staining was performed on paraffin-embedded brain sections (5–10 μm-thick) following previously described standard protocols (Fabian-Fine, Weaver, Roman, Winters and DeWitt, 2024).

### Quantitative analysis

To evaluate the percentage of swell-body stages I-V in both healthy and AD-affected human hippocampus, blinded tissue sections were used to avoid bias. Images of swell-bodies were taken from all areas examined here including the alveus, stratum oriens, and stratum pyramidale. Images of swell-bodies throughout the CA2 and 3 regions were captured and projected onto a ~200 cm × ~110 cm smart board. The swell-body stages were determined and recorded collectively by the participating research team. The group attempted to evaluate comparable numbers of swell-bodies (non-AD: 314; AD: 336). To achieve these comparable swell-body numbers, one additional slice was evaluated for non-AD (4 vs. 3).

A Chi-square test was used to evaluate whether there was a significant association between healthy vs. Alzheimer disease state and the five swell-body stages. Statistics were calculated and the graph was created using Prism 10.5.0 (GraphPad Software).

### Image acquisition

Images of the Luxol H&E, anti-Aβ, and anti-tau immunolabeled paraffin sections were taken using an Olympus compound microscope with digital image acquisition capabilities.

### Extended depth of field

To merge microscopic features across multiple focal depths, images were taken with an Olympus BX60 microscope with motorized stage in focal steps of 0.125–0.5 μm to form an image stack. The stage was computer controlled using Micro-Manager (Edelstein, et al., 2014) and Micro-Magellan (Pinkard, et al., 2016). The image stack was then processed to remove jitter with the Linear Stack Alignment with SIFT rigid transformation (Lowe, 2004), and finally the stack was combined into an output image with the EPFL Extended Depth of Field/Focus (Forster, et al., 2004) plugin [Easy Mode, Quality Setting] in FIJI (Schindelin, et al., 2012).

### Image processing

Confocal images were exported from the ZEN Blue program using the ‘Image export’ function. Pictures and Figures from confocal, histological, and ultrastructural preparations were created using Adobe Photoshop.

## Results

### Hippocampal tanycytes form a vast network of myelinated cell processes that project into the stratum pyramidale

**Figure 1.**
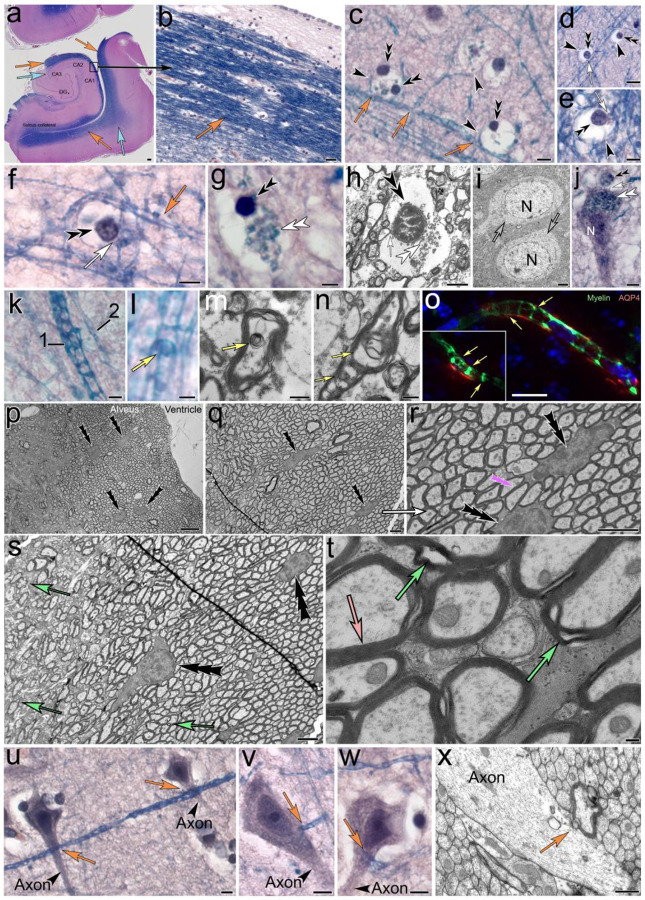
Hippocampal tanycytes form myelinated, varicose cell processes that project into the stratum pyramidale. **(a, b)** Luxol H&E-stained 4-μm section shows the location and appearance of myelinated tanycyte processes (*orange arrows*) in the ventricular lining of the hippocampal formation (*a*). The tanycyte processes (*a, b*) project into the brain parenchyma (*blue arrows in a*). **(c-g)** Individual tanycyte processes give rise to swell-bodies with eletron lucent lumina (*black arrowheads*) that contain one or more nucleus-like tanysomes (*black double arrowheads*). Each tanysyome is physically associated with myelinated tanycyte processes (*white arrows*) .and give rise to receptacles (*white double arrowheads; g, h*). The lumina of swell-bodies appear translucent (*h*) and lack the typical cytoplasmic organelles and structure of cell bodies (*i, mouse hippocampal neurons, N*). **(j)** Tanysome-derived receptacles often project into neuronal somata (*white double arrowheads in h, human hippocampal neuron, N*). **(k-n)** Tanycytes show different swell patterns (k, *1*: swelling process; *2*: normal process). The lumina of tanycyte processes form translucent varicosities that have the appearance of myelinated ring structures (*yellow arrows l*-*n*) that often show increased swelling in AD-affected human brain (*m compared to n*). **(o)** Immunolabeling of mouse brain for Aquaporin-4 (*red*) and myelin (*green*) show similar anti-myelin immunoreactive ring structures in AQP4 double-labeled tanycyte processes. **(p-t)** Ultrastructural depiction of the hippocampal alveus in rat brain shows somata of ependymal tanycytes (*triple arrowheads*) that give rise to vast numbers of varicosity-forming myelinated cell processes that project into the hippocampal formation. The syncytial nature of this tanycyte network is demonstrated by the cytoplasmic connections (*pink triple arrowhead in r*) formed between adjacent somata (*black triple arrowheads in r*). Please note the typical varicose nature of myelinated processes that exit the alveus and project into the hippocampal formation (green arrows in s). Higher magnification shows the myelinated nature of tanycyte processes within the alveus (*light pink arrow in t*) that contain electron-lucent varicosities (*green arrows in t*). **(u-w)** Neuronal axons in Luxol H&E-stained human AD-affected hippocampus lack the varicose nature of the Luxol-stained tanycyte processes that often contact neuronal somata near the axon hillock (*orange arrows*). (u) Similar observations of myelinated, varicose cell profiles (*orange arrow*) transecting into neuronal axons can be observed at the ultrastructural level in rat hippocampus**. *Scale bars:*** *(a)* 500 μm*, (b)* 20 μm*; (c, d)* 10 μm*; (e)* 5 μm*; (f, g)* 5 μm*; (h, i) 2 μm; (j)* 5 μm*; (k) 1 μm, (l) 0.5* μm*; (m, n) 500 nm; (o) 10* μm*; (p) 5* μm*; (q*-*s) 2* μm; *(t)* 100 nm*; (u*-*w)* 5 μm; *(x)* 500 nm.

**Figure 2.**
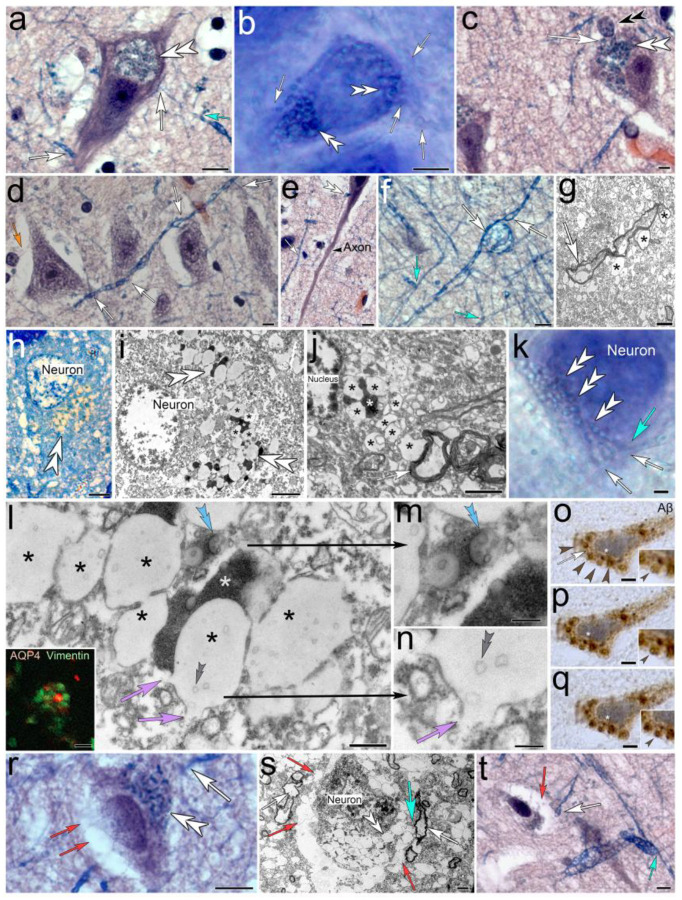
Histological, ultrastructural and immunohistochemical characterization of tanycyte-derived waste receptacles in human hippocampal neurons. **(a)** Neuronal soma in a Luxol blue H&E-stained 10 μm paraffin section that is densely obstructed with abnormally swelling receptacles (*white double arrowheads*). Luxol blue-stained tanycytes processes project into the soma (*white arrows*). Extracellular perpendicular protrusions emerge from the tanycyte process that projects into the neurons (*turquoise arrow*). **(b)** Toluidine-blue stained 70-μm vibratome section demonstrates the magnitude of cytoplasmic obstruction by waste receptacles (*white double arrowheads*) throughout an un-sectioned Alzheimer affected neuron. The hypertrophic, blue-stained receptacles are near translucent tanycyte processes that transect into the soma (*white arrow*). **(c)** Neuronal soma that is contacted by a tanysome (*black double arrowhead*). Receptacles that emerge from the tanysome (*white arrow*) project into the cell soma (*white double arrowhead*). **(d)** A Luxol blue-stained varicose tanycyte process contacts adjacent neurons (*white arrows*). Please note the electron-lucent nature of a tanycyte process that emerges from a tanysome and projects into an adjacent axon hillock (*orange arrow*) **(e-f)** Luxol-blue stained myelinated tanycyte processes form numerous electron-lucent varicosities and larger swell-bodies (*white arrows f, g*) that appear distinctly different from neuronal axons (*e*). The latter lack varicose swellings and appear purple in Luxol blue H&E-stained brain tissue. Please note the varicose perpendicular protrusions that emerge from tanycyte processes (*turquoise arrows in f*). **(h)** Toluidine-blue stained 1-μm semithin-section through a healthy neuron where waste receptacles appear yellowish (*white double arrowhead*). **(i)** Ultrastructural depiction of a healthy neuron with intact, structured cytoplasm and nucleus shows clusters of receptacles that consist of electron-dense compartments (*white asterisk*) that are associated with membrane-bound electron lucent protrusions (*black asterisks*). **(j)** A myelinated tanycyte process (*white arrow*) gives rise to electron lucent varicosities (*black asterisks*) that project into an adjacent cell soma where the receptacles internalize electron-dense material (*white asterisk*). **(k)** Toluidine-blue stained 70-μm vibratome section that contains an un-sectioned Alzheimer affected neuron. The receptacle-formation along perpendicular protrusions (*white double arrowheads*) that emerge (*turquoise arrow*) from an adjacent tanycyte process (*white arrow*) and project into the soma are clearly visible. **(l)** Ultrastructural depiction of waste receptacles in a healthy neuron shows membrane-bound, interconnected electron lucent compartments (*black asterisks*) that have openings to the surrounding cytoplasm (*purple arrows, higher zoom in n*) and contain vesicular structures (*grey double arrowheads*). Associated electron-dense compartments can be seen to internalize vesicular structures through circular openings (*blue double arrowheads, higher zoom in m*). **(o-q)** Different focal planes through an Aβ-immunolabeled neuron in non-AD brain tissue. Serially aligned immunoreactive receptacles (*brown arrowheads in o*) are connected by a cell process (*yellow arrow*). The receptacles contain circular openings that show dark Aβ-immunolabeling (*arrowheads in insets*); *Asterisks*: areas shown in insets. **(r-s)** Light- (*r*) and electron microscopic (*s*) depiction of degenerating neurons shows their dense obstruction with proliferating, swelling waste receptacles (*white double arrowheads*) that emerge from (*turquoise arrow*) associated myelinated tanycyte profiles (*white arrows*). The neuronal cytoplasm and surrounding areas are gradually replaced by spongiform swelling of membrane-bound tanycyte protrusions (*red arrows*). **(t)** Neuron contacted by a swelling tanycyte process (*white arrow*) with advanced cytoplasmic depletion (*red arrow*) with only the neuronal nucleus remaining. An adjacent tanycyte projection that emerges perpendicular from the same tanycyte processes (*turquoise arrow*) shows severe hypertrophic swelling. ***Scale bars:*** *(a)* 10 μm*; (b*-*d) 5* μm*; (e) 10* μm*; (d) 10* μm*; (f) 5* μm *(g) 2 μm, (h) 2.5 μm; (i*-*k) 2 μm; (l) 500 nm, inset 2 μm; (m, n) 200 nm; (o*-*q) 3 μm; (r) 3 μm; (s) 2 μm; (t): 5 μm*

Ependymal tanycytes reside in the ventricular lining of mouse, rat and human hippocampus that borders on the temporal horn of the lateral ventricles. Their long, slender processes form a vast network that projects into the brain parenchyma in the hippocampal formation (Figure 1a, b). Tanycyte processes also project into the brain parenchyma of surrounding areas (Figure 1). The findings on human brain presented in the following were investigated and appeared consistent throughout all areas where tanycyte processes were observed as indicated in Figure 1a. The Luxol blue H&E stain of tanycyte processes is indicative of their myelinated nature (Figure 1). Individual tanycyte processes form electron-lucent ‘swell-bodies,’ each containing between one and six tanycyte-associated nucleus-like organelles in the following referred to as ‘tanysomes’ (Figure 1c–g). Swell-bodies can easily be distinguished from cell-somata, due to their lack of cytoplasmic content or organelles (Figure 1h) typically found in cell bodies (Figure 1g, mouse hippocampal neuron). In contrast to cell nuclei, tanysomes are associated with tanycyte processes (Figure 1e–h) and give rise to receptacles that emanate from (*i*) the tanysome, and (*ii*) surrounding myelinated tanycyte profiles (Figures 1g, h; 2a). These receptacles often project into neuronal somata (Figures 1j, 2). Interestingly, in both human and mouse hippocampus the tanycytes contain myelinated ring structures that swell, thereby enlarging the inner lumina of tanycyte processes in areas where these ‘luminar rings’ are inflated (Figure 1k, l *Luxol blue*-*stain*; Figure 1m, n *ultrastructural depiction*). Immunolabeling shows the aquaporin4- and myelin immunoreactive nature of tanycyte processes and reveals the periodic spacing of these myelin-immunolabeled luminar rings at 3–5 μm intervals (Figure 1o). The varicose appearance of these myelinated processes is consistently observed in mouse, rat, and human brain at both the light- and electron-microscopic levels and distinguishes tanycyte profiles from neuronal axons (Figure 1u–x). As demonstrated at the ultrastructural level in rat hippocampus, ependymal tanycytes are connected through cytoplasmic canals (Figure 1r). This syncytial network sends vast numbers of varicose myelinated cell processes into the underlying hippocampal formation where they form receptacles that project into neurons and surrounding tissue. Interestingly, swelling tanycyte projections are frequently observed contacting neurons at the axon hillock (Figure 1u–w).

### Tanycyte-derived receptacles internalize electron-dense waste within the neuronal cytoplasm

Luxol blue H&E-stained neurons reveal that myelinated tanycytes project into neuronal somata and form numerous circular receptacles (Figure 2). The receptacles either emanate from tanycyte processes directly (Figure 2a, b), or from tanysomes that contact the neuronal soma (Figure 2c). In Alzheimer affected brain both, the receptacles and their associated tanycyte processes show hypertrophic swelling that results in obstruction and gradual depletion of affected neurons and their outer perimeter due to swelling varicosities that emanate from affected tanycytes (Figure 2a–d, r, s). Interestingly, individual tanycyte processes can be seen to contact several adjacent neurons in a serial fashion explaining hypertrophic swelling in adjacent neurons (Figure 2d, see also below). Varicosity-forming, myelinated tanycyte processes can be distinguished from neuronal axons due to their smaller diameters and different staining patterns that are clearly visible in the Luxol H&E-stained preparations (Figure 2e). Neuronal axons lack translucent varicosities that are characteristic to ependymal tanycytes (Figure 2e–g). In non-AD affected toluidine blue-stained semithin sections through human hippocampus the tanycyte-receptacles appear yellow (Figure 2h). Ultrastructural examination of healthy brain shows that this discoloration is due to the internalization of electron-dense material into centrally located repositories. At the subcellular level forming receptacles can be seen to emanate from myelinated tanycyte processes that project into the cytoplasm of adjacent cells (Figure 2j) consistent with our observations in Luxol H&E-stained preparations (Figure 2k). The formation of varicose tanycyte processes that project into a neuronal soma is clearly visible in vibratome sections through AD-affected brain tissue that contains intact neuronal somata (2k). At the ultrastructural level receptacles consist of lateral electron-lucent swellings that have openings to the neuronal cytoplasm and contain internalized vesicular structures (Figure 2l–n). Interestingly, the electron-dense center of waste receptacles contains a circular opening that internalizes vesicular structures that resemble those observed in the surrounding electron-lucent receptacles (Figure 2l, m). Immunolabeling for the tanycyte marker vimentin and AQP4 show vimentin-immunolabeled receptacles that are arranged around a centrally located AQP4-immunoreactive varicosity similar to the ultrastructural appearance of waste receptacles (Figure 2l; inset). Anti-Aβ immunolabeling demonstrates that these intraneuronal receptacles stain for this protein (Figure 2o–q). Interestingly, the Aβ-immunoreactivity along intraneuronal receptacles is observed in healthy neurons and stains centrally located toroid-shaped canal and tubular structures that resemble canal openings (insets in 2o-q). Compared to healthy human neurons Figure 2h–q), degenerating neurons in AD-affected human brain show severe structural degradation (Figure 2r–t). Proliferating and excessively swelling tanycyte protrusions explain the gradual replacement of neuronal cytoplasm by hypertrophic tanycyte processes and swelling waste receptacles (Figure 2r, s). The emergence of swelling waste receptacles from myelinated tanycyte processes that replace the neuronal cytoplasm can be seen at the ultrastructural level (Figure 2s). In advanced degeneration only the neuronal nucleus and some waste receptacles remain, whereas the rest of the neuron appears depleted (Figure 2t).

### Tanycyte-derived swell-bodies differentiate waste-internalizing toroids and receptacles that show strong immunolabeling for anti-Aβ and anti-tau protein.

**Figure 3.**
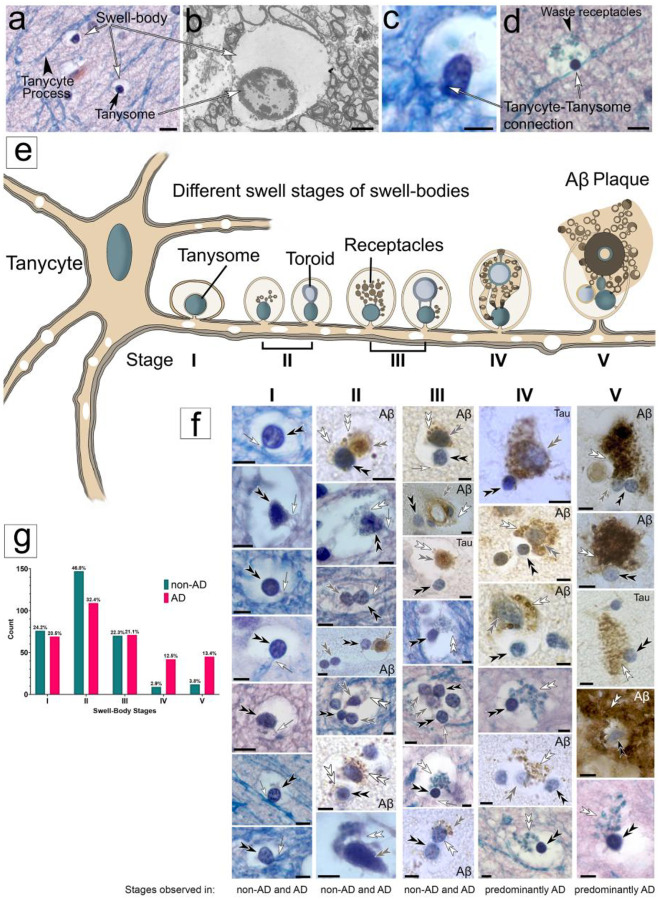
Histological, anatomical, and immunohistochemical characterization of tanycyte-derived swell-body stages in human hippocampus. **(a)** Electron-lucent swell-bodies containing nucleus-like tanysomes form along varicose Luxol-blue stained tanycyte processes in Luxol H&E-stained preparations. **(b)** The electron-lucent nature of the tanysome lumen is apparent at the ultrastructural level. **(b, d)** Luxol H&E-stained swell-bodies with associated tanysomes. Please note the physical connection between tanysome and tanycyte processes (*white arrows*). *Black arrowhead*: Luxol-stained receptacles within swell-body. **(e)** Schematic drawing of tanycyte with swell-bodies that show different stages of receptacle formation (*Stage I*-*V)*. For clarity, only one tanycyte process with swell-bodies is drawn. **(f)** Histological and immunohistochemical images corresponding to the schematic stages shown above each vertical image column. *Stage I* swell-bodies show predominantly clear lumina that contain tanycyte-associated tanysomes; Stage II swell-bodies contain tanysomes that give rise to receptacles or larger toroids (schematic drawing left and right, respectively). Please note the anti-Aβ and anti-tau immunolabeled nature of the toroids and receptacles. In Stage III swell-bodies, both receptacles and toroids swell and detach from the tanysomes, while remaining connected through slender cell processes. Swelling toroids form receptacles around their periphery that are strongly Aβ-immunolabeled. This swelling increases in Stage IV. In Stage V, both toroids and receptacles project out of the swell-bodies into the surrounding tissue or adjacent cells. Both toroids and receptacles show increasingly strong anti-Aβ and anti-tau immunolabeling. *White double arrowheads*: receptacles; *black double arrowheads:* tanysomes*; grey double arrowheads:* toroids*; white arrows:* connective cell processes between tanysome and tanycytes. All histological images are stained for Luxol-H&E. **(g)** The results of the Chi-square test (χ^2^(4, N = 314 [healthy], 336 [Alzheimer]) = 45.75, *p* < 0.0001) indicate a significant association between disease state and swell-body stages. Percentages above the bars indicate the total percentage of a given stage in relation to the total number of evaluated swell-bodies within a given test group (non-AD: N=314; AD: N=336). ***Scale bars:*** *(a)* 10 μm*; (b) 2* μm*; (c) 5* μm*; (d) 10* μm*; (f) 5* μm*. Schematic not drawn to scale*.

**Figure 4.**
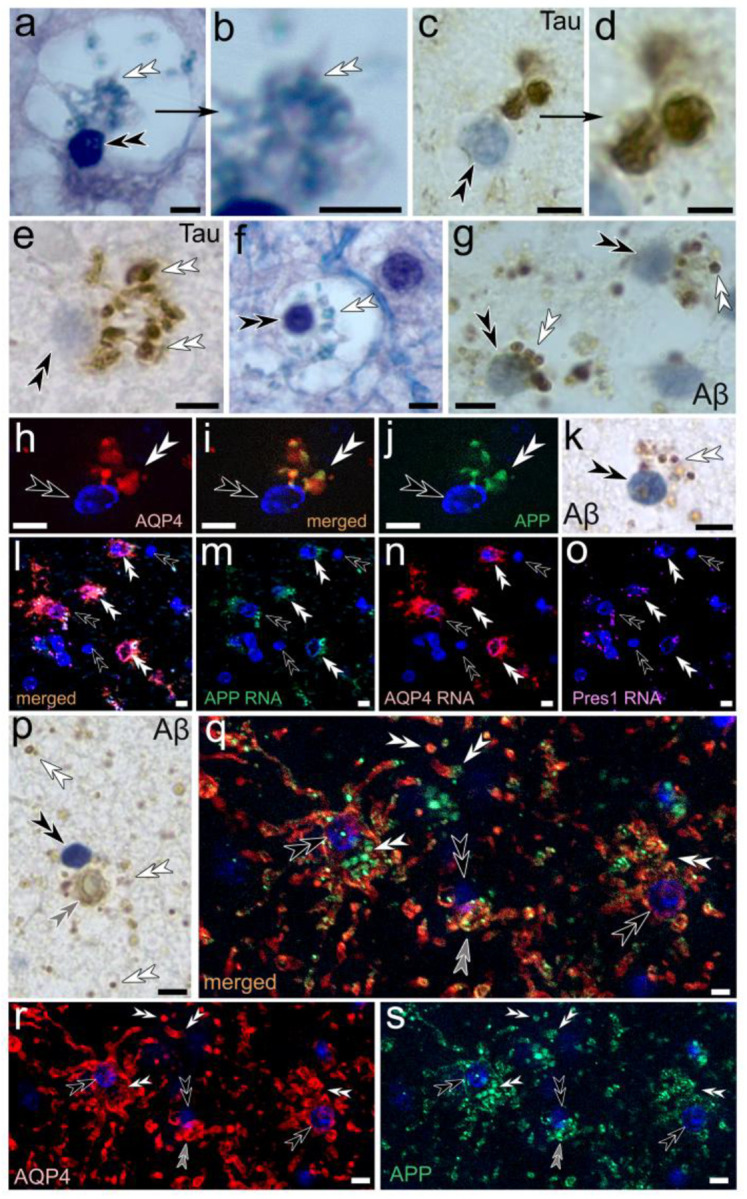
Immunohistochemical characterization of human tanysome-associated waste receptacles in swell-bodies. **(a-g)** Receptacles emanating from tanysomes stain for Luxol blue indicative of myelin (*a, higher zoom in b, f*), anti-hyperphosphorylated tau protein (*c, higher zoom in d, e*), and anti-Aβ (*g*). **(h-o)** Immunolabeling for aquaporin-4 (*h, i*) and amyloid precursor protein (*i, j*) labels receptacles that emanate from tanysomes (*Hoechst blue nuclear stain*). The observed APP-immunolabeling is consistent with the anti-Aβ immunolabeling observed on tanysome-derived receptacles (*k*). **(l-o)** RNA-gene expression for APP (*green*, *l, m*), AQP4 (*red, l, n*) and Presenilin-1 (*magenta*, *l, o*) observed along tanysomes and associated toroids and receptacles are consistent with the proposed formation of Aβ-lined AQP4 expressing receptacles in swell-bodies. **(p-s)** Tanysome-associated receptacles show immunolabeling for Aβ (*p*) and APP (*green*, *q, s*) in AQP4 immunoreactive (*red, q, r*) swell-bodies. Please note restriction of immunoreactivity to tanycyte processes and receptacles and the presence of numerous immunoreactive receptacles outside of swell-bodies that is consistently observed in both Aβ and AQP4/APP immunolabeled human brain of both AD and non-AD-affected hippocampus (*white double arrowheads p, q*). *White double arrowheads*: receptacles; *black double arrowheads:* tanysomes*; grey double arrowheads:* toroids. ***Scale bars:*** *(a)* 5 μm*; (b) 2* μm*; (c) 5* μm*; (d*-*e) 2* μm*; (f*-*s) 5* μm.

Light- and electron-microscopic investigation of swell-bodies in human, non-AD-affected hippocampus shows that each swell-body contains one or more nucleus-like organelles that stain for nuclear stain and are referred to as ‘tanysomes’ (Figure 3). Three primary features distinguish tanysomes from conventional cell nuclei. *(i)* The lumina of swell-bodies are void of cytoplasm and appear electron-lucent at both light and electron-microscopic levels (Figure 3a, b). *(ii)* tanysomes are penetrated by myelinated tanycyte processes (Figure 3c, d) through clearly visible pores that can reach diameters of >2 μm (Figure 3c, see also below). *(iii)* tanysomes give rise to myelin-derived receptacles that internalize electron-dense material consistent with cellular waste (Figures 3; 4; see also below). We have identified five distinct stages of receptacle differentiation in swell-bodies investigating both non-AD and AD-affected brain tissue. *Stage I* swell-bodies are ~6–20 μm in diameter, have electron-lucent lumina and contain one tanycyte-associated tanysome (Figure 3f, *Stage I*). *Stage II* tanysomes give rise to either donut-shaped ‘toroids,’ several smaller receptacles or both. The receptacles stain for Luxol-blue, a histological marker for myelin. Interestingly, both toroids and receptacles also show immunolabeling for anti-Aβ protein in both AD and non-AD-affected hippocampus. In AD-affected tissue, these structures are also labeled for anti-tau protein (Figure 3f, *Stage II*). In *Stage III* swell-bodies, the toroids swell and separate from the tanysomes but remain connected to the tanysomes via thin translucent connections. Increasing numbers of waste receptacles can be observed in individual swell-bodies (Figure 3f, *Stage III*). Both toroids and receptacles continue to increase in size and number in Stage *IV* swell-bodies (Figure 3f, *Stage IV*) and project out of the swell-bodies in Stage *V*. The anti-tau and anti-Aβ immunolabeling observed in AD-affected tissue appears more prominent in Stage *V* compared to Stages I-IV and is consistently associated with both receptacles and toroidal structures (Figure 3f). The results of the Chi-square test (χ2(4, N = 314 [healthy], 336 [Alzheimer]) = 45.75, p < 0.0001) indicate a significant association between disease state and swell-body stages (Figure 3g), although the small number of replicates are only suggestive of a trend that should be further investigated systematically.

### Tanysomes and associated receptacles and toroids express the Aβ-related genes amyloid precursor protein (APP) and presenilin-1 (Pres1) and give rise to Aβ-plaques

**Figure 5.**
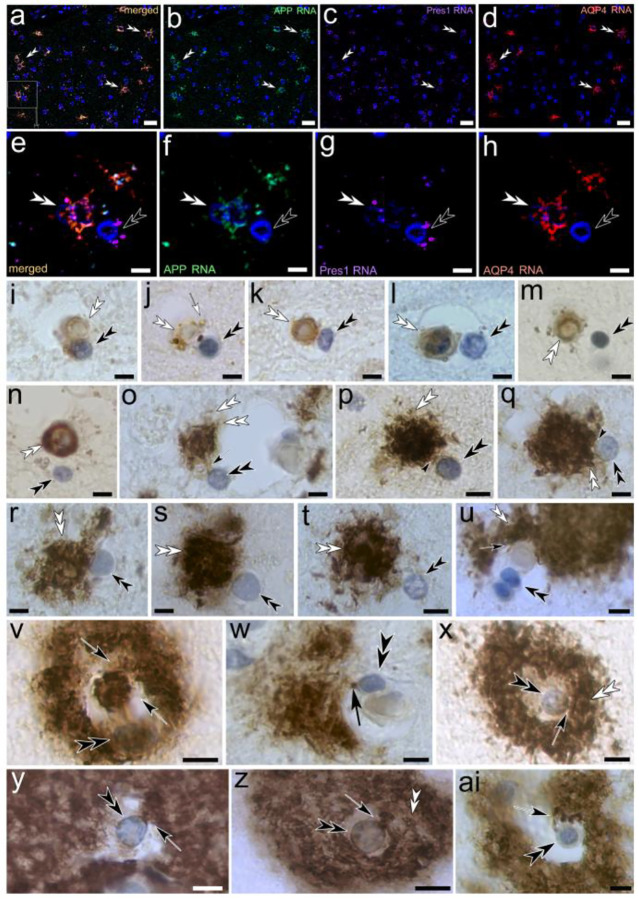
Progressive formation of amyloid beta plaques in AD-affected human hippocampus. **(a-h)** Swell-bodies in human hippocampus show RNA-expression for amyloid precursor protein (*APP, green*), presenilin-1 (*Pres1, magenta*) and aquaporin-4 (*AQP4, red*); *Blue signal*: DAPI nuclear stain, higher zoom shows the gene-expression along tanysomes (*black double arrowheads*) and associated toroids and receptacles (*white double arrowheads)*. **(i-m)** These findings are consistent with the modestly Aβ-immunolabeled nature of swell-body derived toroids and receptacles in AD-unaffected brain tissue that is void of Aβ-plaques. **(n-t)** In AD-affected brain tissue, both receptacles and toroids start to proliferate and swell. **(u-ai)** Investigation of larger Aβ plaques shows the emergence of numerous Aβ-immunolabeled waste receptacles from tanysomes and associated toroids (*black arrows*). **Scale bars:** (a-d) 15 μm; (e-ai) 5 μm.

Immunolabeling of AD-affected human hippocampus for Aβ, anti-Tau protein, the Aβ-related protein APP in addition to the AQP4 water channel demonstrate the association of these proteins with toroids and waste receptacles in swell-bodies (Figure 4). The appearance of immunolabeled swell-bodies and receptacles is consistent in size, shape, and location with Luxol blue H&E-stained preparations (Figure 3). Furthermore, gene expression patterns for APP, Pres1, and AQP4 are consistent with the expression of these proteins in swell-bodies (Figure 4i–o; Figure 5a–h) and support our hypothesis that Aβ and AQP4 may play important functional roles in waste uptake into tanysome-derived waste receptacles (see discussion). Immunolabeling for Aβ (Figure 4p), AQP4, and APP (Figure 4q–s) reveals a vast network of smaller receptacles that emanate from swell-bodies and tanycyte processes (see also below). These smaller immunolabeled sites may easily be dismissed as background labeling. However careful examination of brain sections using confocal microscopy and Differential Interference Contrast microscopy consistently show that these smaller immunolabeled sites that surround swell-bodies depict numerous translucent receptacles whose circular centers are stained for AQP4, APP, and Aβ (Figure 4p–s).

### Progressive formation of Aβ-plaques

**Figure 6:**
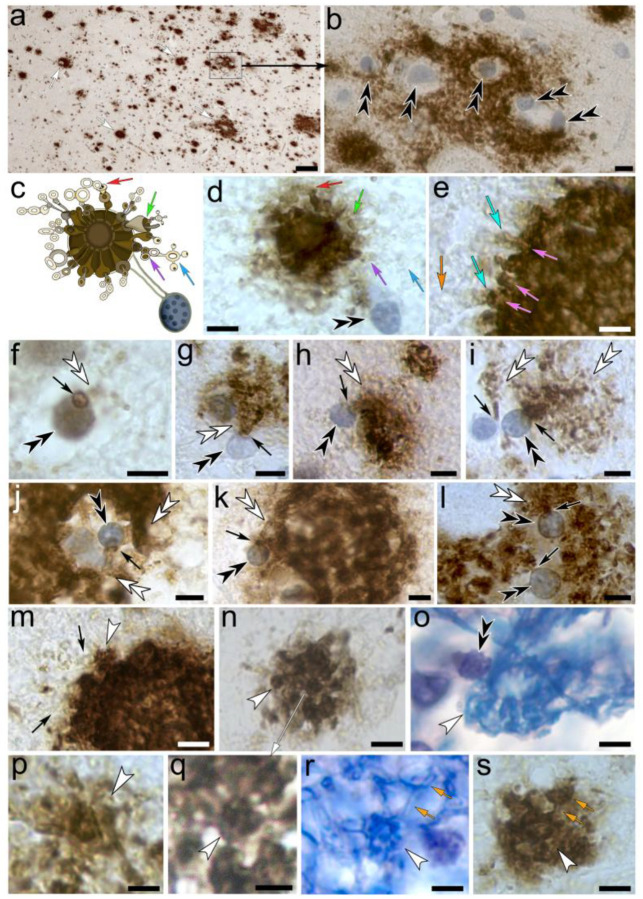
Anatomical features of Aβ plaques in AD-affected human hippocampus. **(a, b)** Dense accumulations of Aβ plaques (*white arrows*) in the hippocampal alveus are associated with tanysome-containing swell-bodies. The boxed area in *a* shown at higher magnification in *b* reveals the accumulation of tanysome-derived Aβ-immunoreactive waste receptacles (*black double arrowheads*). **(c, d)** Schematic drawing of the tanysome-associated toroid in *d* illustrates the emergence of translucent waste receptacles from peripheral canals (*colored arrows show respective areas in c and d*). Note the translucent nature of newly forming receptacles (*blue arrow in d*) **(e)** Partial image of a larger plaque shows strongly Aβ-immunolabelled ‘chimney-like’ peripheral canals (*pink arrows*) from which large numbers of forming translucent receptacles emerge (*turquoise arrows*). Please note the faint Aβ labeling of receptacles that emerge from the plaque (*turquoise arrows*) compared to the unstained nature of more distant receptacles (*orange arrow*). **(f-l)** Tanysomes (*black double arrowheads*) form Aβ-immunolabeled pores (*arrow in f*) from which large numbers of Aβ-immunolabeled toroids and receptacles emerge (*white double arrowheads*) in areas where Aβ plaques are found. **(m, n)** Toroid-shaped plaques consisting of a centrally located toroid from which peripheral receptacles emerge (*white arrowhead*). The latter in turn give rise to receptacles that appear translucent at the light microscopic level (*pink arrows in m*) and contained within a membranous compartment (*pink arrowheads in m*). *Inset:* At the ultrastructural level this membranous compartment is clearly visible (*pink arrowhead*). The toroidal structures contained within the depicted compartment appear electron dense, consistent with internalization of cellular waste (*pink arrow*). **(o)** Tanysome-associated toroid in Luxol H&E-stained human alveus shows a centrally located toroid that contains Luxol-blue stained circular receptacles and ring structures (white arrowhead). (p-s) Higher zoom of star-shaped receptacles (*white arrowheads*) observed in Aβ-plaques and Luxol H&E-stained alveus. Radial extensions from the centrally located toroidal structure form additional receptacles (*orange arrows*). Image in *q* is a higher zoom of the plaque shown in image *n*. ***Scale bars:*** (*a*) 60 μm; (*b, d*-*o*) 5 μm; *inset in m*: 1 μm; (*p*-*s*) 2.5 μm. *Schematic not drawn to scale*.

Figure 5I–Ai demonstrates the progression from Stage II swell-bodies to extremely hypertrophic Stage V swell-bodies in AD-affected Aβ-immunolabeled human brain. *Stage II* swell-bodies (Figure 5i) progressively transition into *Stage III* where the toroids and emanating receptacles separate from their associated tanysomes (Figure 5j–m). As the toroids and associated receptacles continue to swell and transition into *Stages III* and *IV*, the Aβ-immunolabeling appears more prominent (Figure 5n–q). As these swell-bodies progress into *Stage V* (Figure 5r–ai), the areas surrounding affected swell-bodies are densely obstructed with excessively forming Aβ-immunolabeled receptacles that emerge from tanysomes (e.g., Figure 5y) and associated toroids (e.g., Figure 5u, v). *Stages IV* and *V* are particularly abundant in the ventricular lining. Other swell-bodies in AD-affected brain, predominantly in and near the ventricular lining, show increasingly hypertrophic swell-bodies that consist of swelling toroids with forming receptacles around their periphery (Figure 5o–t). The physical association of the Aβ-immunolabeled toroids and receptacles with tanysomes is clearly visible (Figure 5o–q; Figure 6). Larger Aβ-plaques often form around (*i*) several adjacent swell-bodies, or (*ii*) individual swell-bodies that contain multiple tanysomes that are often located in different optical focal planes. In these plaques, large amounts of swelling receptacles can be seen adjacent to or surrounding tanysome-containing swell-bodies (Figure 5u–ai, Figure 6a–m). In Figure 6c, we have provided a schematic drawing of the stage V plaque depicted in Figure 6d to illustrate the formation of smaller, translucent receptacles that emerge form peripheral ‘chimney-like’ canals (corresponding arrows in 6c and d). Figure 6e shows the peripheral area of a larger plaque demonstrating the abundance of small, translucent receptacles that emerge from strongly Aβ-immunolabeled canals. Such peripheral receptacles contain faintly stained smaller receptacles and appear surrounded by a membranous compartment (Figure 6m). These observations are consistent with ultrastructural investigations that demonstrate the electron dense nature of the toroidal structure contained within these peripheral receptacles (inset in 6m, Figure 7) The emergence of translucent waste receptacles is also demonstrated in Figure 7 where we have performed correlative ultrastructural investigations of these receptacles.

### Amyloid β-plaques consist of interconnected star-shaped receptacles that can also be observed in Luxol H&E

High magnification examination of individual Aβ-plaques shows that they consist of star-shaped receptacles each comprised of a central receptacle from which peripheral canal-like extension emanate that interconnect adjacent receptacles (Figure 6k–s). To demonstrate the abundance and consistency by which large amounts of Aβ-immunolabeled, and translucent receptacles emerge from tanysomes and toroids in AD-affected human hippocampus, we have included a larger than usual number of images that show this process (Figures 5–7). Ultrastructural investigations of waste receptacles in AD-affected hippocampus demonstrate the association of both tanysomes and myelinated cell profiles with strands of electron-dense receptacles consistent with the hypothesis that these receptacles internalize cellular waste (Figure 7). Our ultrastructural investigations show the association of numerous waste receptacles with myelinated profiles within and outside of swell-bodies. These observations are consistent with Luxol H&E-stained preparations that show blue coloration of forming receptacles within swell-bodies indicative of myelination (Figure 7j, m).

### Anti-Tau immunolabeling of AD-affected human hippocampus is associated with tanysomes and emanating receptacles and toroids

**Figure 8.**
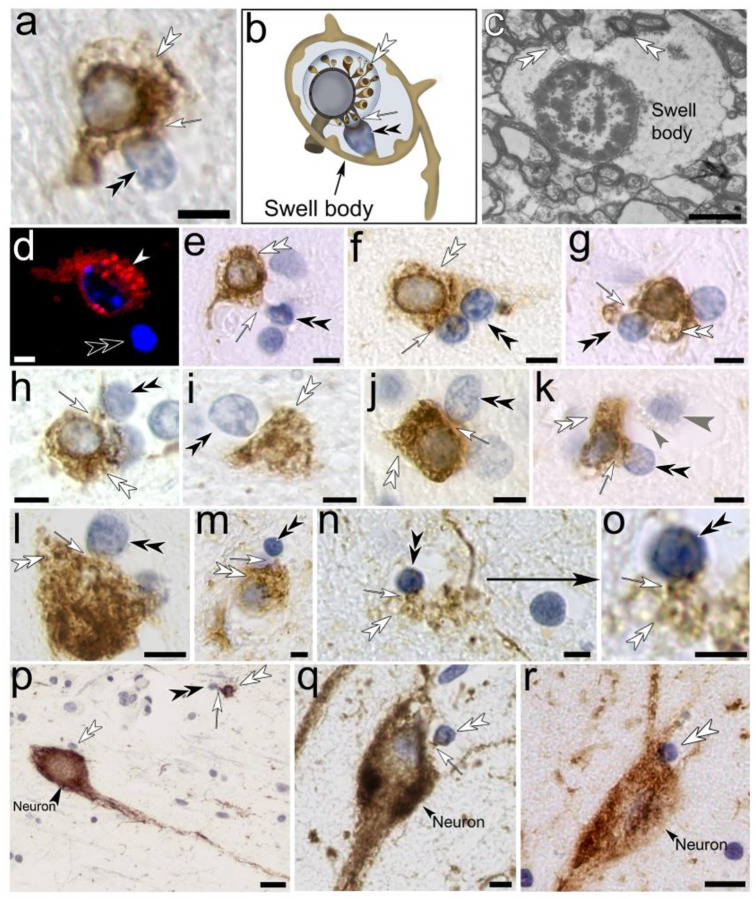
Anti-Tau immunolabeling of AD-affected human hippocampus. **(a)** Swell-body with receptacle-forming toroid shows that the anti-tau labeling is associated with receptacle formation. **(b)** Schematic drawing of swell-body in *a*. **(c)** Electron-micrograph of swell-body with forming myelinated receptacles. **(d)** Swell-body in living mouse hippocampus that was exposed to Cy3 fluorochrome shows brightly fluorescing receptacles consistent with the hypothesis of fluorochrome uptake into receptacles (*white arrowhead*). **(e-o)** Swell-bodies that show the consistency of tau-immunolabeling associated with receptacle formation from both toroids (e, f, g, h, i, j, m) and tanysomes (k, l n with higher zoom in o). Please note the unlabeled swell-body with forming receptacles (*small grey arrowhead*) that protrude from nuclear-stained receptacle shaped structures (*large grey arrowhead*) in k. (p-r) Anti-Tau immunolabeled neurons that are contacted by tanysomes are distinctly larger compared with swell-bodies (*p*). *White double arrowheads*: receptacles; *black double arrowheads: tanysomes, white arrows: connection between tanysomes and receptacles or toroids*. ***Scale bars:*** *(a)* 5 μm; (*c*) 2 μm; (*d*-*o*) 5 μm; (p) 15 μm; (q) 5 μm; (r) 10 μm. *Schematic not drawn to scale*.

Investigation of anti-Tau immunolabeled AD-affected tissue demonstrates that this protein is also associated with swell-bodies and forming receptacles (Figure 8a–o).

Interestingly, the appearance of anti-Tau immunolabeled swell-bodies in human AD-affected brain shows strong resemblance to swell-bodies in living mouse brain that fluoresce after exposure to Cy3 fluorochrome. These preliminary functional observations are consistent with our hypothesis of waste internalization by these structures from their surroundings (Figure 8d). Anti-Tau immunoreactive neurons that are considerably larger compared to immunoreactive swell-bodies (Figure 8p) are often contacted by swell-bodies whose tanysome-derived receptacles project into the neuronal somata (Figure 8q).

### Extended depth of field microscopy visualizes the interconnected, reticular nature of toroid-forming tanycyte processes in AD-affected human hippocampus

**Figure 9.**
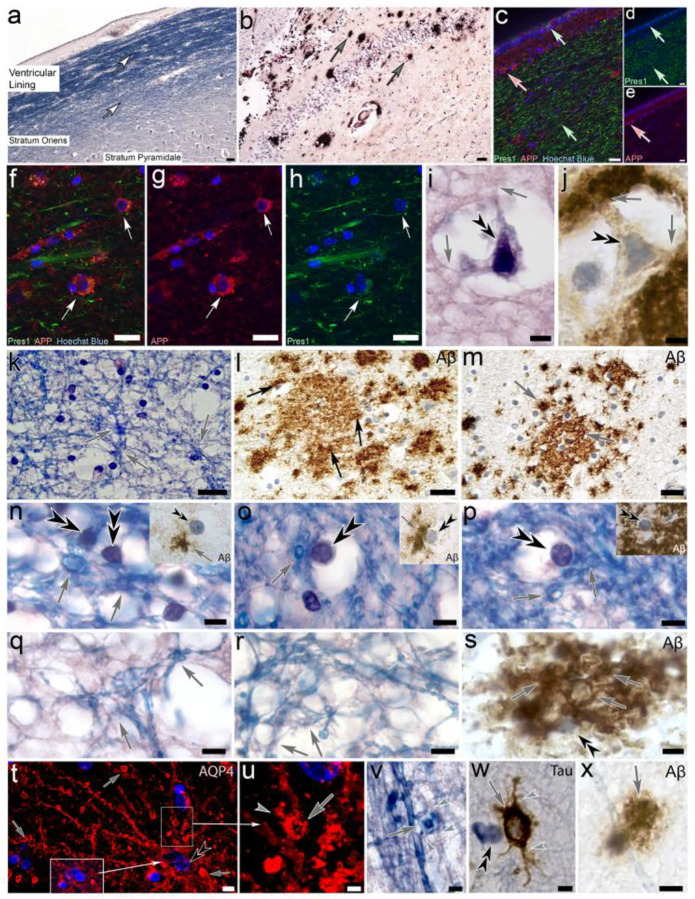
Histological and immunohistochemical depiction of AD-affected human hippocampal alveus and stratum oriens. **(a)** Luxol H&E-stained paraffin section demonstrates the location of myelinated tanycyte processes in the ventricular lining of the anteriomedial hippocampus above the CA3 area (*white arrows*), within the stratum oriens and stratum pyramidale. **(b)** Aβ-immunolabelling demonstrates the accumulation of Aβ-immunoreactive swell-bodies and associated toroids (*grey arrows*). **(c-e)** Immunolabeling of the alveus for presenilin-1 (*Pres1, green, green arrows*) and amyloid precursor protein (*APP, red, red arrows*) demonstrates the expression of both proteins by ependymal tanycytes. **(f-h)** Swell-bodies in the stratum oriens immunolabeled for Pres1 and APP show double labelling associated with tanysome-containing swell-bodies and associated tanycyte processes (*white arrows*). *Nuclear stain: Hoechst blue*. **(i, j)** Two swell-bodies in subsequent Luxol H&E-stained (*i*) and Aβ-immunolabeled (*j*) sections through the same area in the stratum oriens show the formation of toroid-shaped tanycyte processes (*grey arrows*).(k-m) Extended depth of field microscopy in which several focal planes are merged shows the interconnected nature of tanysomes (*black double arrowheads*) with the toroid-forming (*grey arrows*) tanycyte network in both Luxol blue H&E-stained and Aβ-immunolabeled sections. **(n-s)** Luxol blue H&E-stained and Aβ-immunoreactive toroids (*insets in n*-*p*) show the association of tanysomes (*black double arrowheads*) with the surounding toroid-forming (*grey arrows*) network of tanycyte processes. **(t, u)** Aquaporin 4-immunolabeled swell-body that is associated with strongly-immunoreactive, toroid-forning tanycyte processes (*grey arrows, right inset higher zoom in u*). Upregulation of the Hoechst-blue nuclear stain signal demonstrates the presence of three-nuclear stained organelles within the lumen of the swell-body (left *inset in t*). **(v-x)** Swelling toroids (*grey arrows*) that form along tanycyte processes stained for Luxol Blue H&E (*v*), anti-Tau (*w*) and Aβ (*x*). Grey arrowheads: Cellular processes that emanate from the toroids and form an interconnected reticulum of tanycyte processes. ***Scale bars:*** *(a, b)* 30 μm; (*c*-*h*) 20 μm; (*i*-*J) 5 μm; (k*-*m*) 20 μm, (*n*-*t*) 5 μm; (*u*) 4 μm; (*v*) 2 μm, (*w*) 4 μm; (*x*) 3 μm.

Investigation of serial paraffin sections through AD-affected human hippocampus stained for Aβ, Luxol H&E and Tau protein in an alternating fashion demonstrates that Luxol-stained tanycyte processes reside in the ependymal lining and project into the stratum oriens and stratum pyramidale within the anteromedial part of the hippocampal formation (Figure 9a). The same area shows Aβ-immunoreactive structures known as Aβ plaques (Figure 9b). Immunolabeling of this area for the Aβ-related proteins Pres1 and APP show strong labeling for both proteins along tanycyte processes and associated swell-bodies throughout the hippocampus and adjacent brain areas (Figure 9c–h). In Luxol H&E-stained alveus the interconnected nature of this tanycyte network consisting of numerous forming swell-bodies and toroids is clearly visible (Figure 9). This toroid-forming network appears Aβ-immunolabelled, whereby the immunolabeling is consistently associated with tanysome-containing swell-bodies (Figure 9). This interconnected nature is particularly apparent using Extended Depth of Field-Microscopy (Figure 9k–m). Immunolabeling for AQP4, Anti-Tau and Aβ shows that toroids emerging from tanycyte processes are immunolabeled for all three proteins, consistent with our previously published postulation that this cell network is likely formed by AQP4-expressing myelinated tanycytes that shows hypertrophic swelling in AD-affected hippocampus (Figure 9t–x). These observations were not restricted to the hippocampal formation but were consistent throughout all investigated brain areas shown in Figure 1a.

## Summary

**Figure 10.**
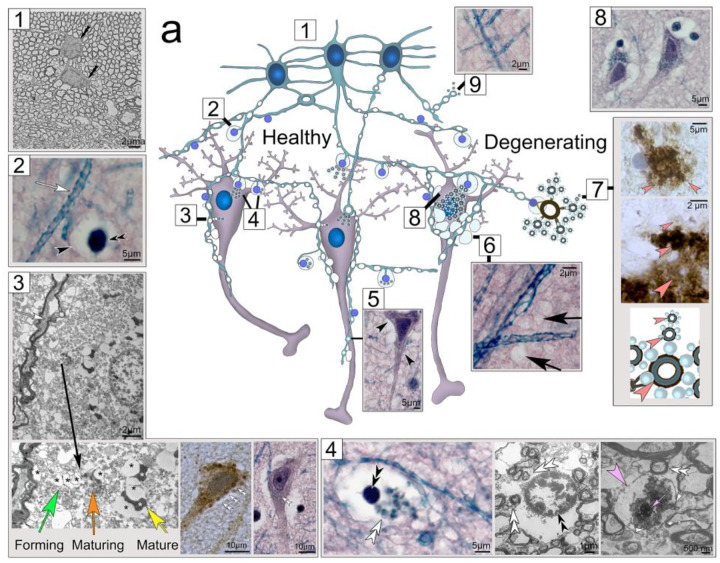
Depiction of the proposed glial canal system in rodent and human hippocampus. **(a)** In healthy brain tissue a vast interconnected syncytium of ependymal tanycytes form long varicose processes into the brain parenchyma (*1*). Tanycyte processes form (*i*) swell-bodies that express aquaporin 4 and amyloid beta related genes (*2*) and (*ii*) varicose protrusions that differentiate into waste-internalizing receptacles (*3*). These receptacles are myelin-derived and project into both neuronal somata and extracellular spaces (*4*). Electron-dense accumulations within waste receptacles are consistent with the appearance of cellular waste (*4*). Tanycyte processes also contact neuronal axons, particularly at the axon hillock (*5*). In Alzheimer-affected brain tanycyte-derived varicosities appear hypertrophic compared to healthy brain tissue leading to an increasingly spongiform appearance of affected brain tissue (*6*). Tanysome-derived waste receptacles proliferate through the emergence of new receptacles from existing receptacles (*orange arrowheads in 7*). The swelling of these receptacles together with their strong Aβ deposits gives them the appearance of Aβ plaques (*7*). Hypertrophic swell-bodies and associated receptacles that transect into neuronal somata cause dense obstruction of affected neurons with swelling receptacles and gradual replacement of cytoplasmic content with swelling, electron lucent tanycyte protrusions (*8*). Spongiform abnormalities of adjacent tissue are caused by the proliferation and swelling of tanycytes and their perpendicular projections (*6* and *9*).

As summarized in Figure 10, the findings presented here are indicative of a vast network of myelinated tanycyte processes that project into the brain parenchyma and internalize intra- and extracellular waste (Figure 10a). We provide ultrastructural evidence that myelin-derived waste receptacles differentiate within (*i*) specialized swell-bodies, or (*ii*) emanate from myelin-derived tanycyte protrusions that project into both neuronal somata and extracellular areas where they mature and internalize electron-dense material consistent with cellular waste. The receptacles are strongly immunoreactive for Aβ and tau protein suggestive of a functional/structural role of these proteins in the waste-uptake process. The presence of APP, Pres1, and AQP4 mRNA within swell-bodies is indicative of protein translation in areas where receptacle formation is observed. We provide strong evidence that hypertrophic swelling of tanycyte processes and associated receptacles in AD affected human brain leads to neuronal obstruction and hypertrophic proliferation of Aβ-lined receptacles that emanate from tanysomes within swell-bodies and have the appearance of Aβ plaques (Figure 10a).

## DISCUSSION

Here, we provide the structural and biochemical foundation that has informed our hypothesis of the existence of a tanycyte-derived glial canal system that internalizes cellular waste from the brain parenchyma. We demonstrate that waste-internalizing receptacles differentiate along tanycyte processes and within swell-bodies where AQP4 and the Aβ-related genes APP and Pres1 are expressed. These findings together with the prominent anti-tau immunoreactivity observed in AD-affected swell-bodies support our postulation that both tau and Aβ proteins may play important roles in the structural and functional architecture of waste receptacles. Moreover, the expression of AQP4 within tanycytes provides a compelling explanation for the different swelling patterns observed along tanycyte processes, swell-bodies and associated receptacles. Our postulation that tanysome-derived receptacles internalize cellular waste is corroborated by our correlative ultrastructural investigations that show the accumulation of electron-dense material within receptacles. Based on the findings presented here we propose an additional functional significance for tanycytes besides their previously proposed metabolic functions (Bolborea and Dale, 2013, Dali, et al., 2023, Ebling and Lewis, 2018, García-Cáceres, et al., 2019, Rodriguez, et al., 2005)

### Evidence in support of the ‘glial canal hypothesis’ and proposed functional model

Based on the findings presented here, we postulate that Aβ-plaques show hypertrophic waste receptacles that emanate from tanycyte-associated swell-bodies. This postulation contradicts the prevalent ‘amyloid hypothesis’ that proposes that Aβ-plaques are due to the accumulation of misfolded Aβ protein (Behl, 2024, Kepp, Robakis, Hoilund-Carlsen, Sensi and Vissel, 2023).

We favor the recently proposed ‘glial canal hypothesis’ (Fabian-Fine, Weaver, Roman, Winters and DeWitt, 2024). Based on the structural and biochemical foundation provided here, we propose the following, testable functional model: waste-internalizing structures require structural stabilization for this process. An analogous anatomical example are cartilaginous rings of trachea that are vital to keep airways open during inspiration (Lambert, et al., 1991, Rains, et al., 1992). We propose that this stabilization may be the functional significance of the luminar rings shown here. As these rings swell, they likely expand and stabilize the inner lumina of the myelinated processes. In this context, the previously proposed circadian dynamic of waste removal (Hablitz, et al., 2020) that may regulate the periodic swelling of these canals provides a compelling mechanism.

*How is waste channeled into the inner receptacles shown here?* This process requires: (*i*) a driving force into the receptacles, (*ii*) openings to the neuronal cytoplasm through which waste can enter the receptacles, (*iii*) stabilization of the receptacle membranes and their openings to prevent their collapse during waste intake, and (*iv*) the dynamic and quantitatively appropriate formation of such waste-internalizing structures. We propose that the driving force is exerted by the expressed AQP4 that is likely located central to the receptacles, thus creating an inward flow into the receptacles. This postulation is supported by the central location of AQP4-immunoreactity in waste receptacle aggregations shown here. We postulate that waste internalization is through receptacle openings shown here at the ultrastructural level. We propose that Aβ may play an important role in the required stabilization of the receptacle membranes and their openings to prevent their collapse. Amyloid β is a lipophilic, structurally stable insoluble protein that is thus uniquely suitable for the stabilizing function of cell membranes (Tycko, 2015). Observations of healthy brain tissue demonstrate that the tanycyte-derived waste receptacles shown here and previously (Fabian-Fine, Weaver, Roman, Winters and DeWitt, 2024) contain structures that are consistent with canal-like openings and are strongly immunoreactive for Aβ. We thus postulate that the observed (lipophilic) Aβ lines these receptacle-membranes to provide the required structural stabilization.

We argue that transport of these proposed functional (AQP4) and structural (Aβ) proteins through narrow tanycytes to areas where dynamic receptacle formation is required would likely be inefficient and risk blockage of narrow tanycyte processes. We propose that these proteins may be translated where receptacle formation takes place, thus explaining the presence of mRNA that encodes for these proteins in swell-bodies where receptacle formation is observed. This proposal is comparable to siding on a house. The components of the siding are made in the production facility (equivalent to mRNA). However, the siding is not assembled at the production facility but rather at the house as it is built. We propose that this is the role of tanysomes in swell-bodies. As demonstrated here and in a previous study, these organelles contain mRNA for AQP4, APP, Pres1 and glial-fibrillary acid protein (Fabian-Fine, Weaver, Roman, Winters and DeWitt, 2024) and likely other key proteins such as Pres2 that remain to be tested. We propose that this mRNA is translated during the formation of waste receptacles and that the functional (AQP4) and structural proteins (Aβ) are incorporated into the forming receptacles (i.e., the siding is assembled around the house where it is built). This proposed model would ensure efficient and dynamic (activity-dependent) formation of waste receptacles where they are needed.

This postulation is based on the observed immunolabeling for the RNA-encoded proteins APP, AQP4, and Pres1 (Fabian-Fine, Roman and Weaver, 2025) together with Aβ along toroids and receptacles that emanate from tanysomes The AQP4-expression in these receptacles explains the swelling nature of tanycyte-derived receptacles, processes, and swell-bodies. Swelling of AQP-expressing cells is well established and would explain the electron-lucent nature and lack of cytoplasmic structure observed in swell-body lumina (Preston, et al., 1992, Wang, et al., 2023). This swelling would furthermore explain why neuronal somata in AD-affected brain tissue are often densely obstructed with receptacles which results in cytoplasmic depletion and cell death (Fabian-Fine, Weaver, Roman, Winters and DeWitt, 2024). Until now, such intraneuronal receptacles have been described as ‘lipofuscin’ that has been proposed to contain toxic waste that remains in neurons indefinitely (Gray and Woulfe, 2005). Considering our findings that myelin-derived tanycytes form receptacles that project into neuronal somata [see also (Fabian-Fine, Roman and Weaver, 2025), we encourage the re-investigation of myelin and myelin-derived structures in the vertebrate brain. The proposed myelin-derived origin of waste receptacles would explain the prominent intraneuronal myelin-specific Luxol-stain observed in neurons of Australian cattle dogs diagnosed with neuronal ceroid lipofuscinosis, a disease that is characterized by progressive neurodegeneration (Schmutz, et al., 2019). We postulate that the structures identified as lipofuscin depict intraneuronal tanycyte receptacles that clear waste from healthy neurons similar to those shown here in human brain. We suggest that pathological swelling of these receptacles may be due to hypertrophic impairment of the AQP4-expressing tanycytes that obstructs neuronal somata resulting in neurodegeneration (Fabian-Fine, Roman and Weaver, 2025, Fabian-Fine, Weaver, Roman, Winters and DeWitt, 2024, Sauvé, et al., 2022). Until now, the concept of an AQP4-mediated convective flow in context of waste removal from the brain has been postulated in context with astrocytes and the proposed glymphatic system (Iliff, et al., 2012).

### Proposed functional significance of tau protein

In context of the proposed waste removal system introduced here we argue that an additional structural requirement for this glial canal system is ‘dynamic adaptability.’ To ensure adequate waste removal, the quantitatively appropriate release of waste receptacles is imperative. Too many receptacles pose the risk of neuronal obstruction and depletion; too few receptacles may cause waste buildup within the neurons. Previous investigations on spider brain have shown that neurodegeneration is preceded by pathological unraveling of adjacent myelinated glial cells (Fabian-Fine, Weaver, Roman, Winters and DeWitt, 2024). As a consequence, in areas where the glial cells unravel, we have observed excessive formation of waste-internalizing glial canals that lead to the catastrophic depletion of affected neurons (Fabian-Fine, et al., 2023, Fabian-Fine, Weaver, Roman, Winters and DeWitt, 2024). Interestingly, in this arachnid model system, the forming glial canals are anchored to microtubules. This observation led us to propose that the dynamic instability of microtubules is a uniquely suitable mechanism to enzymatically and dynamically control the quantitatively appropriate release of waste-internalizing glial canals. Triggering mechanisms may include mechanical or biochemical signals such as increasing hydrogen concentrations caused by lysosomal activity that produces cellular waste (Fabian-Fine, Weaver, Roman, Winters and DeWitt, 2024). Interestingly, tau protein is well known to stabilize microtubules in the mammalian brain (Barbier, et al., 2019). As demonstrated here, its hyperphosphorylated form is abundant in AD-affected swell-bodies in which excessive receptacle proliferation is observed. It is thus feasible that microtubules may play a similar role in controlling the quantitatively appropriate release of waste receptacles and that this mechanism may fail in AD. This idea would explain the increased formation of waste receptacles that coincides with immunolabeling for hyperphosphorylated tau protein shown here.

### Tanycytes and their proposed roles

Tanycytes are ependymal glial cells that have predominantly been described along the ventricular lining of the third and fourth ventricles and are currently subdivided into four major types based on their location alpha-1 and −2, beta-1 and −2. However, the presence of additional tanycyte types has been proposed (Fabian-Fine, Weaver, Roman, Winters and DeWitt, 2024, Pasquettaz, et al., 2021). These ependymal glial cells that label for glial fibrillary acid protein and vimentin send long, slender processes into the hypothalamic parenchyma and are proposed to have metabolic functions (Bolborea and Dale, 2013, Bolborea, et al., 2020, García-Cáceres, Balland, Prevot, Luquet, Woods, Koch, Horvath, Yi, Chowen and Verkhratsky, 2019). Interestingly, the formation of varicose protrusions along tanycytes that contact neurons have been observed in the hypothalamus of mice (Pasquettaz, Kolotuev, Rohrbach, Gouelle, Pellerin and Langlet, 2021). The degradation of tanycytes in AD-affected brain has previously been reported (Sauvé, Ternier, Dewisme, Lebouvier, Dupré, Danis, Rasika, Kim, Ciofi, Giacobini, Buée, Landrieu, Pasquier, Maurage, Nogueiras, Schwaninger and Prevot, 2022). The authors propose that tanycyte impairment may play a prominent role in neurodegeneration. Based on our findings here, we concur with this postulation.

## Conclusions

This study provides novel insight into the histopathology of AD-affected brain tissue and offers a new hypothesis for the formation of Aβ plaques and neurofibrillary tau tangles and intraneuronal obstructions with waste-containing receptacles. We are acutely aware that we are unable to address other important topics in this study. This includes: (*i*) the striking resemblance of brain structure identified as astrocytes in brain disease (Verkhratsky, et al., 2023) with *Stage III*-*V* swell-bodies shown here, (*ii*) the roles of myelin and glial fibrillary acid protein in the brain, (*iii*) the possible role of tanycytes with regard to gliomas, (*iv*) possible biochemical pathways that trigger receptacle formation in swell-bodies, and (*v*) underlying causes that lead to excessive receptacle formation. We have addressed the latter to some degree here and in previous publications where we propose a link between AD and possible viral, fungal, and bacterial infections whose impact is increasingly recognized (Muzambi, et al., 2020). It is feasible that structural proteins of such pathogens are not sufficiently catabolized prior to uptake into the glial canal system and may lead to blockage (Fabian-Fine, Roman and Weaver, 2025, Fabian-Fine, Weaver, Roman, Winters and DeWitt, 2024). The resulting increase in intracellular turgor would explain the excessive release of receptacles from these AQP4-expressing organelles. These and other topics must be addressed in more detail in future studies. We concur with previously voiced suggestions to critically re-evaluate the ‘amyloid hypothesis’ (Kepp, Robakis, Hoilund-Carlsen, Sensi and Vissel, 2023) and consider alternative hypotheses, one of which is presented here.

## Figures and Tables

**Figure 7.** Correlative light and electron-microscopic characterization of tanysome-derived waste receptacles in AD-affected human hippocampus. **(a)** Amyloid β-immunolabeled swell-body with receptacle-forming tanysome. Please note the strong circular immunolabeling in the center of forming receptacles. **(b)** Ultrastructural depiction of a swell-body with centrally located tanysome and waste receptacles. Some receptacles are filled with electron-dense material (*white arrowheads*) while others appear less electron-dense (*beige arrowhead*). Receptacles also emanate from myelinated tanycyte profiles that border the outer periphery of the swell-body (*blue arrowhead*) and can be seen to form connections to both tanysomes and surrounding myelinated cell profiles (*green arrowheads*). **(c, d)** Tanysome with associated Aβ-immunolabeled toroid (outlined by grey double arrowheads) from which lateral strands of waste receptacles emanate (white double arrowhead, higher zoom in d). **(e)** Luxol H&E-stained tanycytes in the alveus show Luxol-stained receptacles emanating from tanysomes. **(f-i)** Amyloid β-immunolabeled toroids from which translucent waste receptacles emanate (*black arrows, g and I depict higher zooms of images f and h respectively*). Please note the strong immunolabeling of the pore from which receptacles emanate in *f, g (white arrows)*. **(j, k)** Tanysome-associated toroids with emanating waste receptacles. **(l-n)** Higher zoom of peripheral waste receptacles (*l*) indicated in *k* show emanating peripheral receptacles similar to the one shown in Luxol H&E-stained swell-body. Correlative electron-microscopy depicts a myelinated receptacle associated with electron-dense material consistent with internalization of cellular waste. **(o-t)** Electron-micrographs of waste-internalizing receptacles that emanate from myelinated tanycyte profiles. *White double arrowheads*: receptacles; *black double arrowheads: tanysomes; grey double arrowhead: toroid; black arrowhead: areas in which electron*-*dense waste receptacles emanate from myelinated tanycyte profiles or tanysomes*. ***Scale bars:*** *(a)* 5 μm*; (b)* μm*; (c*-*h) 5* μm*; (i) 1* μm*; (j, k) 5* μm; *(l)* 2 μm; *(m) 5* μm; *(n*-*t)* 500 nm.

## Data Availability

All data presented in this study are available from the first author upon request.
